# Crossed beaks in a local Swiss chicken breed

**DOI:** 10.1186/s12917-018-1398-z

**Published:** 2018-03-05

**Authors:** Sara Joller, Flurina Bertschinger, Erwin Kump, Astrid Spiri, Alois von Rotz, Daniela Schweizer-Gorgas, Cord Drögemüller, Christine Flury

**Affiliations:** 1 Institute of Genetics, Vetsuisse Faculty, University of Bern, Bern, Switzerland; 2 School of Agricultural, Forest and Food Sciences, Bern University of Applied Sciences, Zollikofen, Switzerland; 3 ProSpecieRara, Basel, Switzerland; 4 Züchterverein für ursprüngliches Nutzgeflügel, Neukirch an der Thur, Switzerland; 5 Divison of Veterinary Anatomy, Vetsuisse Faculty, University of Bern, Bern, Switzerland; 6 Division of Clinical Radiology, Vetsuisse Faculty, University of Bern, Bern, Switzerland

**Keywords:** Beak deformity, Indigenous breed, Congenital anomaly, *LOC426217*, GWAS

## Abstract

**Background:**

Crossed beaks have been reported to occur in Appenzeller Barthuhn, a local Swiss chicken breed. The assumed causes for this beak deformity which are also seen in other bird species including domestic chickens, range from environmental influences to genetic factors. The aim of this project was to characterize the prevalence, the phenotype, and the underlying genetics of crossed beaks in Appenzeller Barthuhn chickens.

**Results:**

The estimated prevalence of 7% crossed beaks in Appenzeller Barthuhn was significantly higher compared to two other local Swiss chicken breeds. A breeding trial showed significantly higher prevalence of offspring with deformed beaks from mating of affected parents compared to mating of non-affected parents.

Examination of 77 Appenzeller Barthuhn chickens with crossed beaks showed a variable phenotype presentation. The deviation of the beak from the median plane through the head ranged from 1° to 61°. In more than 60% of the cases, the upper and lower beak were bent in the same direction, whereas the remaining cases showed different forms of crossed beaks. Computed tomographic scans and bone maceration of the head of two chickens with crossed beaks revealed that the maxilla and the mandibula were affected, while other parts of the skull appeared to be normal.

The gene *LOC426217*, a member of the keratin family, was postulated as a candidate gene for beak deformity in domestic chickens. Sequencing of the coding region revealed two significantly associated synonymous variants for crossed beaks in Appenzeller Barthuhn chickens. A genome-wide association study and a comparative analysis of runs of homozygosity based on high-density SNP array genotyping data of 53 cases and 102 controls showed no evidence of association.

**Conclusions:**

The findings suggest a hereditary cause of crossed beaks in Appenzeller Barthuhn chickens. However, the observed variation in the phenotype, together with the inconclusive molecular genetic results indicates the need for additional research to unravel the genetic architecture of this beak deformity.

**Electronic supplementary material:**

The online version of this article (10.1186/s12917-018-1398-z) contains supplementary material, which is available to authorized users.

## Background

A bird’s beak is a defining feature of its head. It is important for the intake of food and water [1, 2], for grooming purposes and parasite removal [3–5] and as a tactile sensory organ [6]. The form of a beak is very well adapted to the trophic niche of its species [7, 8]. It is evident that divergence from a normally shaped beak poses a great problem for the bird. A well-known example is the red crossbill (*Loxia curvirostra*) showing distinctive mandibles, crossed at the tips, which enable them to extract seeds from conifer cones and other fruits [9]. A bird’s beak consists of the osseous maxilla and mandibula, which are covered by a horny sheath of keratin (rhamphotheca). The rhamphotheca is essentially a modified epidermis, where cells of the stratum corneum contain tricalcium phosphate, hydroxyapatite and keratin, giving the beak its rigidity [10]. The rhamphotheca continues growing throughout life and maintains its intended form through continuous wear and tear [6, 11]. In chickens, two nares (nostrils are found laterally at the base of the upper beak which are partially covered by the operculum nasale [12]).

In domestic chickens, beak deformities affect welfare and production. The occurrence of different types of crossed beaks was already reported almost a century ago in various breeds [13, 14]. The crossed beak is a beak deformity that can be defined as misalignment of the upper and lower beak [11]. Until today, there are few reports on prevalence of crossed beaks in chicken [11, 14]. It was estimated that 0.32% of all wild birds have crossed beaks [11]. In a recent study, prevalence ranging from 1 to 3% was documented for local Chinese chicken breeds [15, 16].

Different reasons for the occurrence of beak deformities in birds have been proposed: they can result from the underlying bony structure, from a malocclusion of the mandibles or disturbances in the stratum germinativum of the epidermis [10, 17]. The occurrence of crossed beaks was reported due to breeding conditions [18], accidents or traumata [19], abnormal abrasion of the rhamphotheca [11], housing conditions [8], environmental influences such as toxins [20–24] or nutritional deficiencies [25, 26], infections [27, 28], or genetic causes [14, 23, 29–32]. Already in 1934 a simple recessive inheritance for crossed beaks was postulated in the budgerigar [33]. In 1938 Landauer [14] failed to establish a pure line of domestic chicken with crossed beaks in spite of massive inbreeding. He concluded that the phenomenon is caused by several genes with incomplete penetrance. Several possible candidate genes for deformed beaks in a Beijing-You chicken were proposed from digital gene expression profiling studies [15]. Most of the identified genes belonged to the keratin family, as well as genes coding for proteins important in the biosynthesis of unsaturated fatty acids and the metabolism of glycerolipids. A follow-up study on the most promising gene *LOC426217* found five variants that differed significantly between chickens with crossed beaks and normal controls [16].

**Fig. 1:**
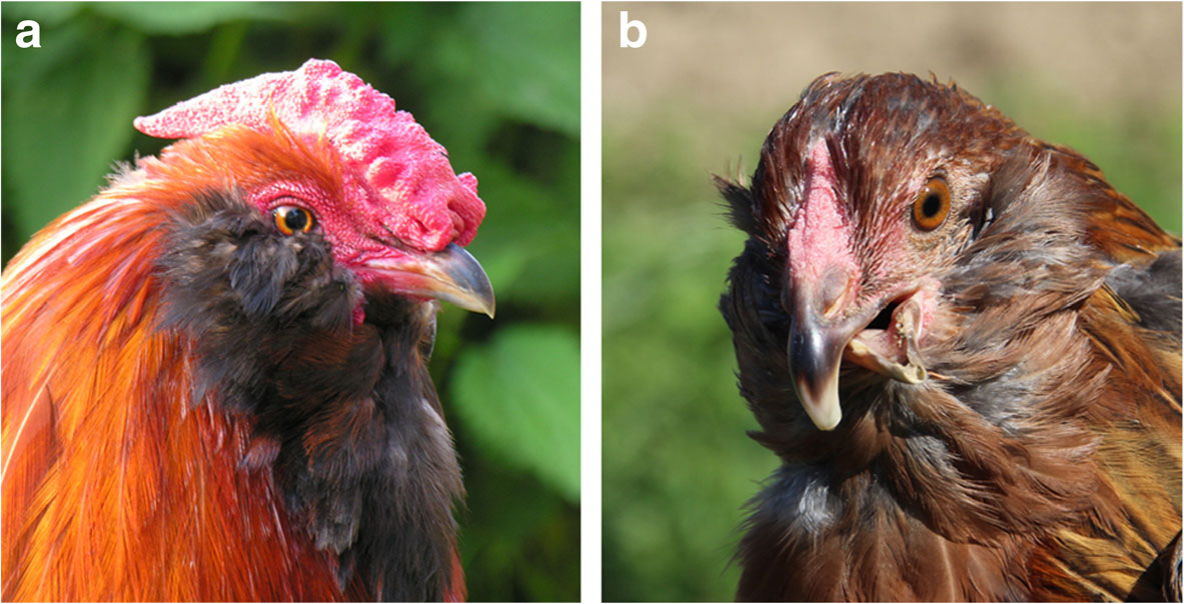
Normal and crossed beak: (**a**) Appenzeller Barthuhn rooster with a normal beak and (**b**) Appenzeller Barthuhn hen with a crossed beak

In recent years, breeders have occasionally reported the occurrence of crossed beaks in Appenzeller Barthuhn chickens (Fig. 1). Along with the Appenzeller Spitzhaubenhuhn and the Schweizerhuhn, this breed represents one of three local chicken breeds found in Switzerland [34]. The aim of this study is to describe the crossed beak phenotype based on Appenzeller Barthuhn cases and to derive estimates of prevalence using data from a survey and a breeding trial. First attempts to unravel the genetic architecture of the crossed beak phenomenon are presented.

## Methods

### Breeding analysis

A total of 165 breeding reports, provided by breeders, were analyzed to derive the prevalence of crossed beaks for the breeding seasons 2012–2017. Breeders reported their annual breeding success and the number of hatched animals showing crossed beaks. Estimated prevalence were compared by Pearson’s chi-squared test with Yates’ continuity correction using the R software [35].

### Breeding experiment

Animals from the 2016 breeding season were selected based on the shape of their beak at 12 weeks of age, and included in a targeted breeding experiment as the parental generation. This comprised in total of six breeding units of comparable size: three units consisting of affected animals and three units consisting of control animals (Additional file 1). In the three affected units, three to four affected hens and one affected rooster were grouped together in each unit, resulting in 11 affected hens and three affected roosters (Additional file 1). The three control units consisted of five hens and one rooster each, totaling 15 hens and three roosters with a normal beak. The six parental breeding units were grouped in August 2016 and were kept constantly under the same feeding regime and environmental conditions until the 2017 breeding season.

The F1-generation eggs of the six parental groups were collected during three periods of 10 days in spring 2017, and incubated under standardized conditions in a conventional hatchery. After hatching, chicks were individually tagged and reared until 12 weeks of age. The occurrence of beak deformities was visually controlled, and the final status was assessed between 10 and 12 weeks of age. Proportions of affected and unaffected offspring were compared using Fisher’s exact test in the RStudio software [35].

### Phenotype description

Between the years 2012 and 2017, a total of 77 chickens with complete, undamaged heads, displaying supposed beak deformities were presented by breeders of Swiss Appenzeller Barthuhn chickens and collected from the breeding trial. All heads were photographed and macroscopically examined for 11 morphological features (Additional file 2). In addition, the angles of the upper and lower beak were measured. The head was fixed with the beak in a horizontal position between two lateral and one caudal screw and a photo was taken from directly above. Based on the photograph, the 0° axis was defined as perpendicular to the two screws and through the median plane of the head (Additional file 3). The axis of the beak was defined by drawing a line along the base and middle part of the upper or lower beak, while ignoring all additional bending of the tip. The angle was then measured at the intersection of 0° axis and the axis of the beak (Additional file 2). Statistical analyses were conducted with the software RStudio software [35]. Angles of the upper beak, lower beak and in between beaks were tested for normal distribution with Shapiro-Wilk normality test. One sample t-test for normal distributed data and Wilcoxon signed rank test for non-normal distributed data were used respectively, to test whether the beaks were bent more to one side than the other. The Wilcoxon signed rank test was used to compare angles of upper and lower beaks. The binomial test was used to examine association between bending of the beak and bending of the tip. A detailed anatomical examination of two cases with crossed beaks and one normal Appenzeller Barthuhn control were carried out by computed tomography and maceration of the head bones (10% aqueous Biozyme SE-Solution, 60 °C, 20 h).

### Candidate gene analysis

The annotated exon of the candidate gene *LOC426217* on chromosome 25 was sequenced in 53 affected Appenzeller Barthuhn chickens (specified in Additional file 4) and 114 breed controls. The DNA was isolated either from EDTA-blood or tissue samples using the Nucleon Bacc2 kit (GE Healthcare). In addition, DNA samples of 42 Appenzeller Spitzhaubenhuhn and 42 Schweizerhuhn were analyzed for comparison. PCR amplification was done as previously described [16] and subsequently sequenced by direct Sanger sequencing on the 3730 DNA-Analyzer (Thermofisher). The obtained sequence data was analyzed with Sequencher 5.1 software (Gene Codes). For all variants allele and genotype frequencies were compared between cases and controls using chi-squared and Fisher’s exact tests. The Benjamini and Hochberg method [36] was used for multiple testing correction of *p*-values. Phased haplotypes were considered for further analysis.

### Genome-wide analysis

Genomic DNA samples from 53 cases and 102 control Appenzeller Barthuhn chickens were genotyped with the Axiom™ Genome-Wide Chicken Array Kit (Affymetrix) for 580,961 SNP markers. PLINK v1.07 [37] was applied for quality control by removing: SNPs with a call rate below 99%, SNPs with a minor allele frequency below 1% and significant (*p* ≤ 0.0001) deviation from the Hardy-Weinberg equilibrium. Additionally, genotypes of individuals with a call rate below 90% were excluded. In total, 155 individual genotypes consisting of 341,115 SNPs remained for the genome-wide association study (GWAS). A mixed linear model was fitted with the GenABEL package [38], including a random polygenic effect based on an autosomal genomic relationship matrix, in order to account for population structure and relatedness between animals. SNPs were considered to be genome-wide significantly associated if their *p*-values were below the 5% Bonferroni-corrected threshold for 341,115 independent tests (pBONF 1.47 × 10^− 7^). Runs of homozygosity (ROH) were detected using PLINK v1.90 [37] with a density of at least 1 SNP per 85 kb. The percentages of animals having a SNP in a ROH were compared between cases and controls at all the different loci on all chromosomes.

## Results

### The prevalence of crossed beaks differs between breeds

Across the three Swiss local chicken breeds, a total of 78 out of 2698 chicken (2.9%) were reported to show a crossed beak within the past six years (Table 1). The anomaly occurred in all three breeds, but the prevalence differs. The Appenzeller Barthuhn had a significantly higher prevalence (7.4%) compared to Appenzeller Spitzhaubenhuhn (1.5%, χ2 = 18.726, *p*-value < 0.001) and Schweizerhuhn (0.8%, χ2 = 70.351, *p*-value < 0.001) (Table 1). No significant difference was observed in the prevalence between Appenzeller Spitzhaubenhuhn and Schweizerhuhn (χ2 = 1.1262, *p*-value = 0.2886).

**Table 1:** Occurrence of crossed beaks in three local chicken breeds based on breeder reports between 2012 and 2017

Breed	Number of reports	Total number of birds	Normal	Crossed beak
Appenzeller Barthuhn	55	797	738 (92.6%)	59 (7.4%)
Appenzeller Spitzhaubenhuhn	35	454	447 (98.5%)	7 (1.5%)
Schweizerhuhn	75	1447	1435 (99.2%)	12 (0.8%)
Total	165	2698	2620 (97.1%)	78 (2.9%)

In the F1-generation of the breeding trial, 83 offspring of parents with crossed beaks and 102 offspring of parents with normal beaks were examined between 10 and 12 weeks of age (Additional file 1).

A significant difference in prevalence (*p*-value = 0.003) of crossed beak offspring was observed when comparing these two groups: 2.9% of the offspring from normal parents showed crossed beaks, whereas 15.7% affected animals were observed in the offspring of parents with crossed beaks (Table 2). Additionally, seven animals (3.8%) displayed deformed but not crossed beaks. This fraction did not differ significantly between the two parental groups (Table 2).

**Table 2:** Number of offspring with and without crossed beaks from affected and unaffected parents observed during the breeding experiment

	Number of offspring with normal beak (fraction in %)	Number of offspring with crossed beak (fraction in %)	Number of offspring with otherwise deformed beak (fraction in %)
Parents with crossed beaks	67 (80.7%)	13 (15.7%)	3 (3.6%)
Parents with normal beaks	95 (93.1%)	3 (2.9%)	4 (3.9%)
Total	162 (87.6%)	16 (8.6%)	7 (3.8%)

### The crossed beak anomaly in Appenzeller Barthuhn chicken

**Fig. 2:**
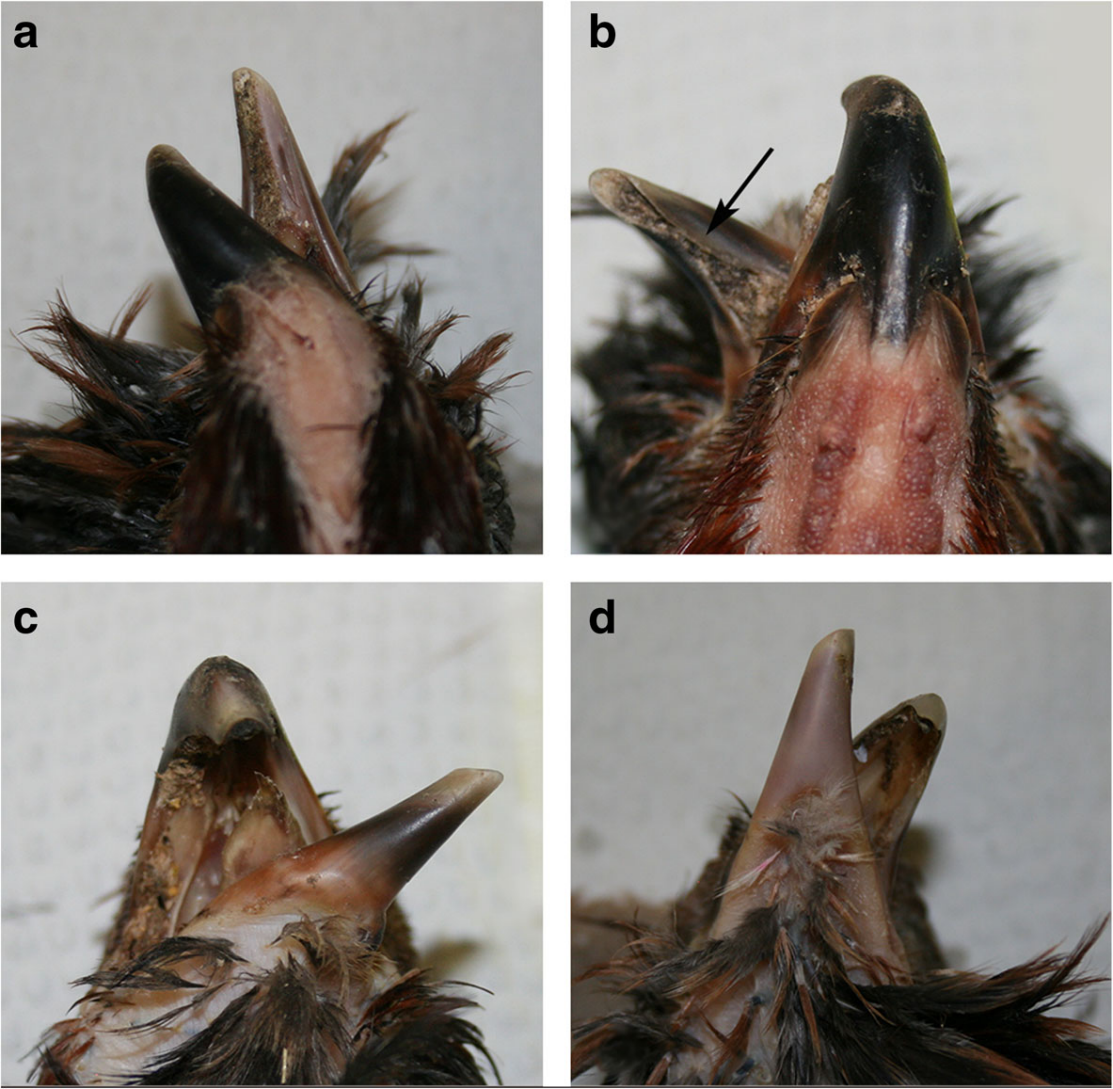
Examples of typical crossed beak in Appenzeller Barthuhn chickens. Note that the beaks are bent in different directions and at a different location**. a** Dorsal view on an upper beak bent to the left at the base of the beak. **b** Dorsal view of an upper beak bent only at the tip, while the lower beak is bent at the base with an inwardly-rolled tomium. **c** Ventral view of a lower beak bent at the base. **d** Ventral view of a lower beak bent at the tip and an upper beak bent at the base

The crossed beak is characterized by one or both beaks deviating laterally from the longitudinal axis of the head. Out of 77 cases collected from breeders and the breeding trial, 48 (62.3%) showed an upper and lower beak that deviated clearly either left or right (Fig. 2), while in 5 cases (6.5%) the upper and lower beak deviated on different sides. Of the 48 cases where both beaks deviated in the same direction, 44 (91.7%) showed a stronger bending of one of the two beaks. In 23 of 77 cases (29.9%) the upper beak was straight while the lower beak was bent. A bent upper beak without a bent lower beak occurred only in one case (1.3%).

Upper and lower beaks which deviated from the median plane showed a wide range of angles from only 1° up to 50° in the upper beak and from 2° up to 61° in the lower beak. About 50% of all deviation angles of beaks were between 1° and 9° for the upper beak, and 2° and 12° for the lower beak, respectively. The upper beak was more often bent to the right side (*p*-value = 0.029), while no significant difference in the direction of the lateral bend of the lower beak (*p*-value = 0.905) could be found. There was no significant difference between the angles of the upper and the lower beak (*p*-value = 0.5822). The angle between upper and lower beak varied between 0° (cases where both upper and lower beak deviated at exactly the same angle) and 61°.

**Fig. 3:**
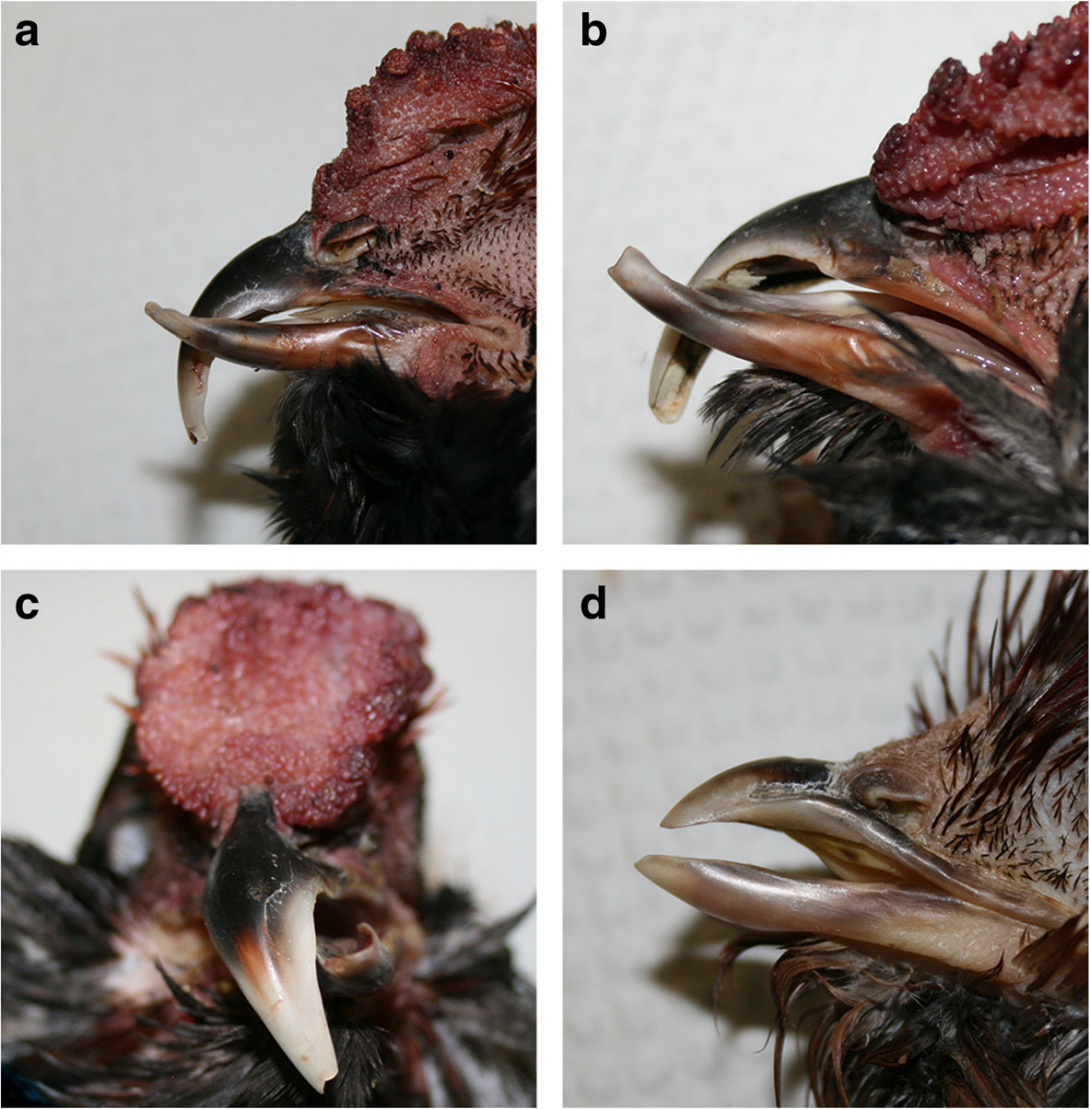
Occasionally observed additional features in Appenzeller Barthuhn chickens with crossed beaks. **a + b** The upper beak bent excessively downwards, while the lower beak is bent upwards. Note the overgrowth of both beaks. **c** The beak is not only bent horizontally, but is also rotated along its longitudinal axis. **d** The upper beak is bent upwards at its base

In 51 out of 77 cases (66.2%), one or both tips of the beak were turned in the direction of the other tip, as if trying to find its counterpart (Fig. 2b, c). This was independent of the original bending of the beak. Out of 77 beaks available for examination, 47 (61.0%) showed a rotation of the beak (Fig. 3c). The direction of the rotation was associated with the direction of the bending of the beak; it usually rotated in the same direction as the bending (*p*-value = 0.002).

A total of 64 cases (83.1%) showed an excessively vertically downward bent upper beak. In 21 cases (27.3%), the lower beak was bent upwards and in 21 cases (27.3%) bent downwards (Fig. 3a, b). In 23 cases (29.9%) the upper beak of an affected animal was also bent upwards at the base, usually accompanied by a protruding of the palate (Fig. 3d). Out of 77 cases examined, 47 (61.0%) showed one or both beaks elongated (Fig. 3a, b). Both beaks were elongated in 31 cases (40.3%), only the upper beak in nine cases (11.7%) and in seven cases (9.1%) only the lower beak. In nine cases (11.7%) the upper beak was too short compared to a normal beak and in three (3.9%) of these cases, the short upper beak was paired with an elongated lower beak.

**Fig. 4:**
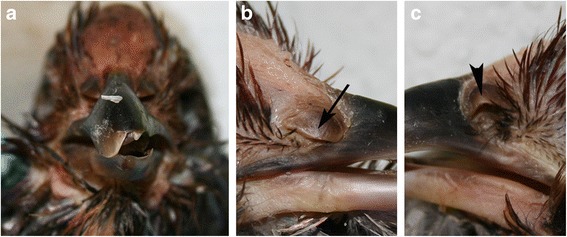
Occasionally observed anomalies of the rhamphotheca and nares in Appenzeller Barthuhn chickens with crossed beaks. **a** Overgrowth of the beak resulting in an instable brittle tip. **b + c** Deformed nares occur due to the bending of the upper beak at the height of the nares. Note that the nostril on the convex side of the beak was nearly entirely closed due to a stretched operculum, while the operculum on the concave side was folded

The rhamphotheca in animals with crossed beak showed no overall abnormality. Most commonly observed were brittle tips on overlong beaks, sometimes with pieces of tip missing (Fig. 4a). The normal beak of a domestic chicken has a sharp-cut tomium, however in 11 birds (14.3%), the tomium was rolled inwards (Fig. 2b). In 51 cases (66.2%) food and soil got stuck in one or both beaks, mostly in a part of the beak that was not covered by its counterpart. This was promoted by an inward rotation of the tomium (Fig. 2b).

The remainder of the head including the cranium, eyes, ears, feathers and wattles did not show any outward abnormality. The most rostral part of the rose comb was often bent with the upper beak. The nares, situated laterally and at the base of the upper beak were in some cases compressed on one side and stretched on the other side depending on the bending of the upper beak (Fig. 4b, c). The operculum on the convex side of the bending was stretched and entirely covered the naris (Fig. 4b), while the operculum on the concave side was folded and left the nostril wide open (Fig. 4c).

### A detailed view on the crossed beak phenotype in Appenzeller Barthuhn chickens

**Fig. 5:**
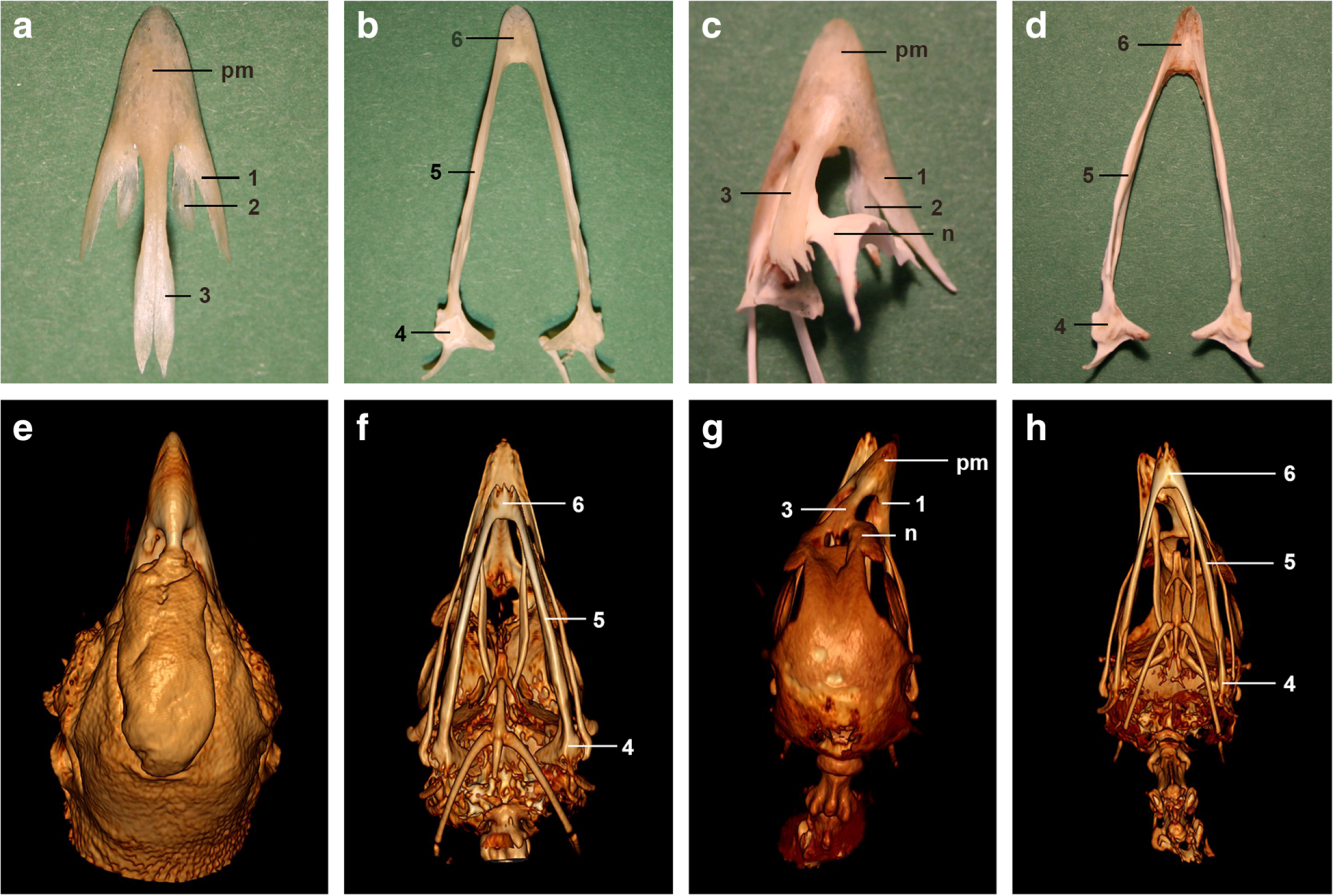
Comparative view of the head of a normal Appenzeller Barthuhn chicken with an animal with crossed beaks. Macerated bones of the beak of a control chicken (**a + b**) and a chicken with crossed beak (**c + d**). Volume rendered three-dimensional computed tomographic model of the normal head of a control (**e + f**) and of a crossed beak (**g + h**): view from dorsal (**e + g**) and ventral (**f + h**). Note that both upper and lower beak of the crossed beak are bent to the right. Os praemaxillare (pm) of the maxilla with processus maxillaris (1), processus palatinus (2), processus frontalis (3), and the os nasale (n). Mandibula with pars caudalis (4), pars intermedia (5), and pars symphysialis (6)

Maceration and computed tomographic images showed that the shape of several bones was affected by the crossed beak (Fig. 5c–h) compared to normally formed beaks (Fig. 5a–f). In both examined cases, the rostral part of the skull was asymmetrical. The os praemaxillare and the os nasale were bent to the right in both cases and the beak was rotated around the longitudinal axis. The beak’s tip was bent to the left. In both animals the mandibula also showed a flexion (Fig. 5d). The convex side of the pars intermedia was longer than the concave side (Fig. 5d, h). The pars caudalis of the mandibula was symmetrical (Fig. 5h). Computed tomographic transverse and dorsal reformatted images of affected heads showed the location of the bending. In the upper beak, the bending occurred at the height of the aboral end of the processus frontalis of the os praemaxillare and the nostrils (Fig. 5g). The more rostral part of the beak seemed straight and without inflection, except the tip, which was bent in the opposite direction. The mandibula was bent on the whole length of the pars intermedia, while the base of the mandibula, on the height of the pars caudalis, was still symmetrical (Fig. 5h).

### Associated *LOC426217* variants in crossed beaks

Sequencing of *LOC426217* in 53 cases (of upper and lower beak deformity) and 114 controls revealed a total of 12 variants (Table 3). A single variant was located in the 5′-untranslated region, whereas the remaining variants were in the coding region, including 4 missense variants and 7 synonymous variants. There was a significant genotype frequency difference between cases and controls at two synonymous coding variants (Table 3). Subsequently, a haplotype estimation using the genotypes of these 12 variants in all animals was performed, which revealed three predominantly occurring *LOC426217* haplotypes (Table 4). In addition, further rare haplotypes occurring in seven animals were observed. Based on the diplotypes for these 160 animals carrying the three most frequent haplotypes, a significant (*p*-value < 0.001) difference in haplotype distribution between cases and controls was observed (Table 4). However, the most abundant haplotype A observed in Appenzeller Barthuhn chickens with crossed beaks was also by far the most common haplotype in the genotyped cohorts of animals belonging to the two other local Swiss breeds (Table 4, Additional file 5).

**Table 3:** Genotype frequencies for *LOC426217* variants in normal Appenzeller Barthuhn chickens compared to animals with crossed beaks

Position in the *LOC426217* cDNA^a^ and on chromosome 25 (Gallus_gallus-5.0)^b^		Genotype^c, d^			χ^2^-value^e^	*p*-value	corrected *p*-value
		AA	AB	BB			
c.-62G > T; 5’UTR g.2013308C > A	control case	7 0	26 6	81 46	6.960	0.029	0.086
c.16C > G; p.Leu6Val g.2013231G > C	control case	113 52	1 0	0 0	none	1	1
c.24 T > C; synonymous g.2013223A > G	control case	17 5	56 21	41 26	3.087	0.214	0.320
c.36G > C; synonymous g.2013211C > G	control case	35 10	59 17	20 25	16.849	< 0.001	0.003
c.48A > G; synonymous g.2013199 T > C	control case	82 46	26 5	7 1	5.538	0.063	0.103
c.49G > A; p.Ala17Thrr g.2013198C > T	control case	111 52	3 0	0 0	none	0.553	0.603
c.96C > T; synonymous g.2013151G > A	control case	82 47	25 6	7 0	6.762	0.034	0.078
c.222 T > C; synonymous g.2013025A > G	control case	17 5	56 22	41 26	2.819	0.244	0.326
c.252A > G; synonymous g.2012995 T > C	control case	82 48	25 5	7 0	8.013	0.018	0.068
c.256_258delCTGinsTAT; p.Leu86Ile g.2012991_2012989delGACinsATA	control case	82 47	25 6	7 0	6.762	0.034	0.078
c.289G > T; p.Gly97Cys g.2012958C > A	control case	114 52	0 1	0 0	none	0.317	0.381
c.363 T > C; synonymous g.2012884A > G	control case	21 25	58 18	35 10	15.011	< 0.001	0.003

^a^NCBI accession no. XM_423880

^b^NCBI accession no. NC_006112

^c^For some variants individual genotypes are missing (Additional file 5)

^d^A = reference allele, B = variant allele

^e^None = for these variants fisher test was used and no χ^2^-value exists

**Table 4:** Distribution of diplotypes at *LOC426217* in chickens with and without crossed beaks, and in two other local Swiss chicken breeds

Diplotypes for estimated *LOC426217 haplotypes*	Appenzeller Barthuhn		Appenzeller Spitzhaubenhuhn	Schweizerhuhn
	Control	Case		
AA	20	24	40	41
AB	43	17	1	
BB	17	5		
BC	11	5		
CC	7	0		
AC	11	0		
other	5	2	1	1
Total	114	53	42	42

### Crossed beak phenotype shows no evidence of genetic association at the genome-wide level

The GWAS based on high-density SNP array genotyping data of 53 cases and 102 controls from the Appenzeller Barthuhn breed showed no evidence for association (Additional file 6). Additional GWAS testing of 20 cases showing mostly upper or 31 cases showing mostly lower beak deviation against all controls also revealed no evidence for association. A comparative analysis of runs of homozygosity was performed using the SNP genotyping data of all 53 cases and 102 controls. No major difference was observed between groups (Additional file 7).

## Discussion

Within the last century, the sporadic occurrence of the crossed beak phenotype has been described by various authors in wildlife as well as in domestic birds including chickens [13, 15, 39, 40]. Pomeroy [11] defined a crossed beak as the upper or lower beak deviating from the median plane through the head. There are birds where this naturally occurs, such as the red crossbill (*Loxia curvirostra*). The upper and lower beaks of the crossbill are aligned when they hatch and cross with growth [14]. This development has also been reported in domestic chicks with a crossed beak [14]. The same holds for the offspring produced by the breeding experiment conducted here: the beak deformities were not present at hatching. Nonetheless, in some of the cases presented by breeders, chicks already had a crossed beak at a few days old. The onset of crossed beaks in the affected animals therefore varies. Based on the 185 offspring evaluated in the breeding experiment, it can be concluded that chickens should not be labelled free of crossed beaks before the age of at least 12 weeks.

This study reports for the first time a detailed phenotype characterization of crossed beak in chickens based on morphological measurements and anatomical examination. Nearly 80 years ago Landauer [14] developed a classification of crossed beaks based upon descriptive observations. The first two categories included crossed beaks accompanied by abnormalities of the eyes or the skull. This was not observed in our study, as none of the examined affected Appenzeller Barthuhn chickens had other obvious anomalies of the head. The third category comprised of crossed beaks that developed after 1 to 2 months of age, while the fourth category consisted of crossed beak present at hatching but later grow into a normally developed beak. As indicated above, the crossed beak phenotype in the Appenzeller Barthuhn chickens unambiguously manifested after two months of age and we therefore conclude that the third category is of major concern in this local breed. Landauer [14] also described that the affected beak was usually the maxilla and that the crossed beak had its origin in a malformed bone, not only a malformed rhamphotheca. The cases described in this current study demonstrated both upper and lower beaks were affected.This partially confirms the observations of Landauer [23], as the crossed beak phenotype in Appenzeller Barthuhn clearly affects the bones of the beak. Contrary to this in wild birds, crossed beaks often seem to consist of an abnormally formed rhamphotheca over normally formed bones e.g. avian keratin disorder [11, 40, 41]. Nevertheless, there are reports of cases of affected wild birds, where the bone is clearly malformed as well [31]. One feature in the presented cases of crossed beaks in Appenzeller Barthuhn chickens is very similar to the avian keratin disorder, i.e. the overgrowth of the rhamphotheca. This might be due to the absence of abrasion because of the different use of the beak in domestic chicken and the missing conjunction of upper and lower beak. Previously it has been suggested that upper and lower beak influence each other’s growth during development [11]. It may be due to such influences that traits like tips bent in the direction of the other tip, up and downwards bent tips or rotation of the beak develop. It could be speculated that this may be the result of physiological mechanisms trying to revert the missing conjunction of upper and lower beak in growth [42].

As hypothesized earlier a genetic cause is responsible for the development of crossed beaks in chickens. A significantly higher prevalence of affected animals was found in the Appenzeller Barthuhn breed compared with two other local chicken breeds. This information combined with the known differences in the breeds [43] suggests a major variation in the genetic background for this phenomenon in the Appenzeller Barthuhn chicken. In addition, the results of the targeted mating experiments further support the inheritance of this anomaly. By comparing status of offspring from normal parents with those from affected parents, a significantly higher number of affected offspring resulted from parental units with affected animals. Nonetheless, the occasional occurrence of affected offspring in the control units and the quite high number of non-affected offspring from affected parental units does not allow elucidation of the mode of inheritance.

The gene *LOC426217* located on chromosome 25 was recently proposed as a functional candidate for crossed beak [15, 16], therefore, allele- and haplotype frequencies based on 166 samples (53 affected and 114 controls) of the Appenzeller Barthuhn breed were investigated. From the five variants proposed by Bai et al. [16], three were not polymorphic in Appenzeller Barthuhn chickens. However, seven additional variants, mostly predicted to have no effect on the encoded amino acid sequence are presented. A comparison of the genotype frequencies revealed two variants with significant differences between cases and controls. These two variants seem to confirm the findings of Bai et al. [16] for *LOC426217* as a possible candidate gene for crossed beak, although the phenotype of that study included only lower beak deviation. As both associated variants do not cause a change in the amino acid sequence, their functional impact on the phenotype expression is not obvious. Furthermore, the subsequent haplotype association analysis also showed a significant difference between cases and controls in Appenzeller Barthuhn chickens. As the associated *LOC426217* haplotype also occurs almost exclusively in the genotyped control cohorts of Appenzeller Spitzhaubenhuhn and Schweizerhuhn, it is unlikely that these variants explain the crossed beak phenotype. It could be speculated that other DNA variants, e.g. structural variants affecting larger segments of DNA, situated in the flanking region containing regulatory important elements of *LOC426217* might be present on the associated haplotype in Appenzeller Barthuhn only which could lead to the development of crossed beaks. On the other hand, the identification of *LOC426217* as a candidate gene for beak deformities is based on transcript analysis from lower mandibles [16]. This gene belongs to the keratin family, and it could be concluded that the initially observed difference in expression represents a secondary effect due to the existence of the deviated lower beak.

Finally, the GWAS and the analysis of runs of homozygosity did not support the association with any specific region of the chicken genome. However, the breeding analysis suggests a genetic background. The previously identified single locus association for the tested *LOC426217* variants on chromosome 25 was not confirmed by the genome-wide study. The SNP array marker density was most likely sufficient so it could be assumed, that the previous result might be false positive. The current findings suggest that the crossed beak anomaly in Appenzeller Barthuhn chickens cannot be explained by simple Mendelian inheritance as was previously assumed [33]. The collection of 53 cases used for GWAS shows an obvious variable phenotype expression supporting a complex genetic background. Therefore it is concluded, that a case-control design and the available sample sizes are not sufficient to unravel the genetic architecture of the crossed beak. Additional studies using more genotypes of affected and unaffected animals with a clear description of the phenotype are therefore required.

## Conclusion

Phenotypic variability in chickens with crossed beaks exists. Genetic predisposition in the Appenzeller Barthuhn chicken breed is evident. The targeted mating experiments confirmed a genetic influence on the occurrence of crossed beak chickens. Genetic analyses were inconclusive, indicating a complex mode of inheritance.

## Additional files

Additional file 1: Scheme of the breeding trial. In total six breeding units of comparable size, made up the parental generation: three units consisting of affected animals (orange units, three to four affected hens and one affected rooster) and three units consisting of control animals (blue units, five hens and one rooster). For the F1-generation, eggs of the six parental groups were collected and incubated. After hatching, chicks were reared until the age of 12 weeks. (TIFF 1025 kb)

Additional file 2: Phenotype details of Appenzeller Barthuhn chickens with crossed beak and all cases used in the study. (XLSX 19 kb)

Additional file 3: Phenotyping tool for the measurement of beak angles. The head is fixed between two screws, which sit in the orbita of the skull, and one caudal screw, which supports the caudal part of the head. The photograph is taken from directly above. Based on the photograph, the 0° axis is defined as perpendicular to the two screws and through the median plane of the head (central vertical line of the grid). The axis of the beak is defined by drawing a line along the base and middle part of the upper or lower beak (yellow line). (TIFF 10326 kb)

Additional file 4: Table with all cases for phenotype description and genetic analyses. (XLSX 12 kb)

Additional file 5: Genotypes and diplotypes of Appenzeller Barthuhn and two other Swiss Chicken breeds. Genotypes of 12 variants and their diplotypes in *LOC426217* of Appenzeller Barthuhn, Appenzeller Spitzhaubenhuhn and Schweizerhuhn. (XLSX 30 kb)

Additional file 6: GWAS of 53 cases and 102 controls. (A) Manhattan plot. The red line marks the 5% Bonferroni-corrected threshold for 341,115 independent tests (pBONF 1.47 × 10^− 7^). (B) MDS plot. (C) QQ plot. (TIFF 1480 kb)

Additional file 7: Comparison of runs of homozygosity between cases and controls on the autosomes. Chromosome-wise plots show on the x-axis the mega base (Mb) position on the chromosome (CHR), and on the y-axis the proportions of cases (yellow) and controls (black) being homozygous. (TIFF 1394 kb)

## Abbreviations

Bp: Base pairs
GWAS: Genome-wide association study
Kb: Kilo base pairs
Mb: Mega base pairs
PCR: Polymerase chain reaction
ROH: Run of homozygosity
SNP: Single nucleotide polymorphism
