# Supplementary figures and images for: Crossed beaks in a local Swiss chicken breed
Source: BMC Vet Res. 2018 Mar 5;14:68. doi: 10.1186/s12917-018-1398-z (PMC5838925; doi:10.1186/s12917-018-1398-z)

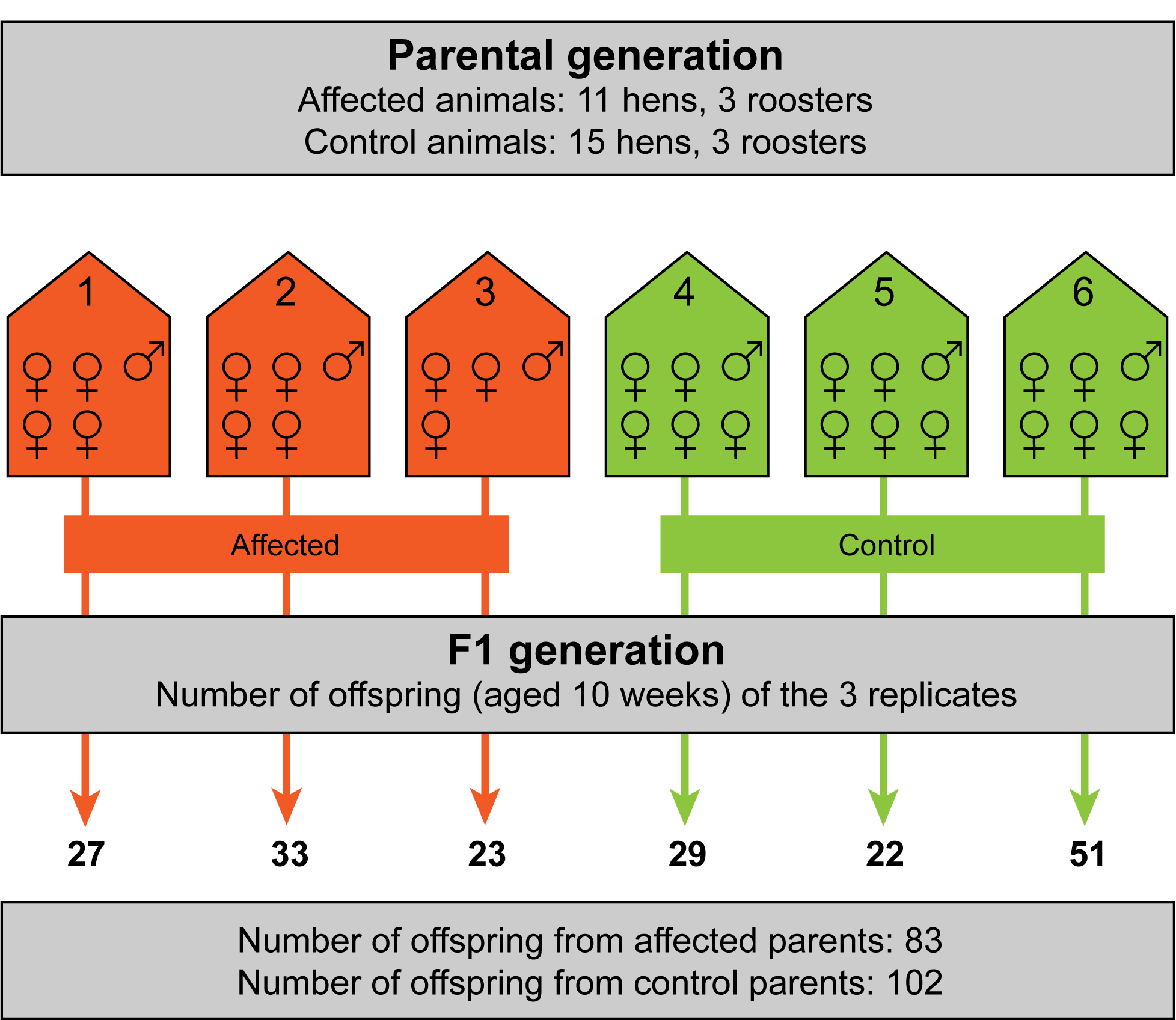

Supplement: Supplementary file 1 — Scheme of the breeding trial. In total six breeding units of comparable size, made up the parental generation: three units consisting of affected animals (orange units, three to four affected hens and one affected rooster) and three units consisting of control animals (blue units, five hens and one rooster). For the F1-generation, eggs of the six parental groups were collected and incubated. After hatching, chicks were reared until the age of 12 weeks. (TIFF 1025 kb) [file 12917_2018_1398_MOESM1_ESM.tif]

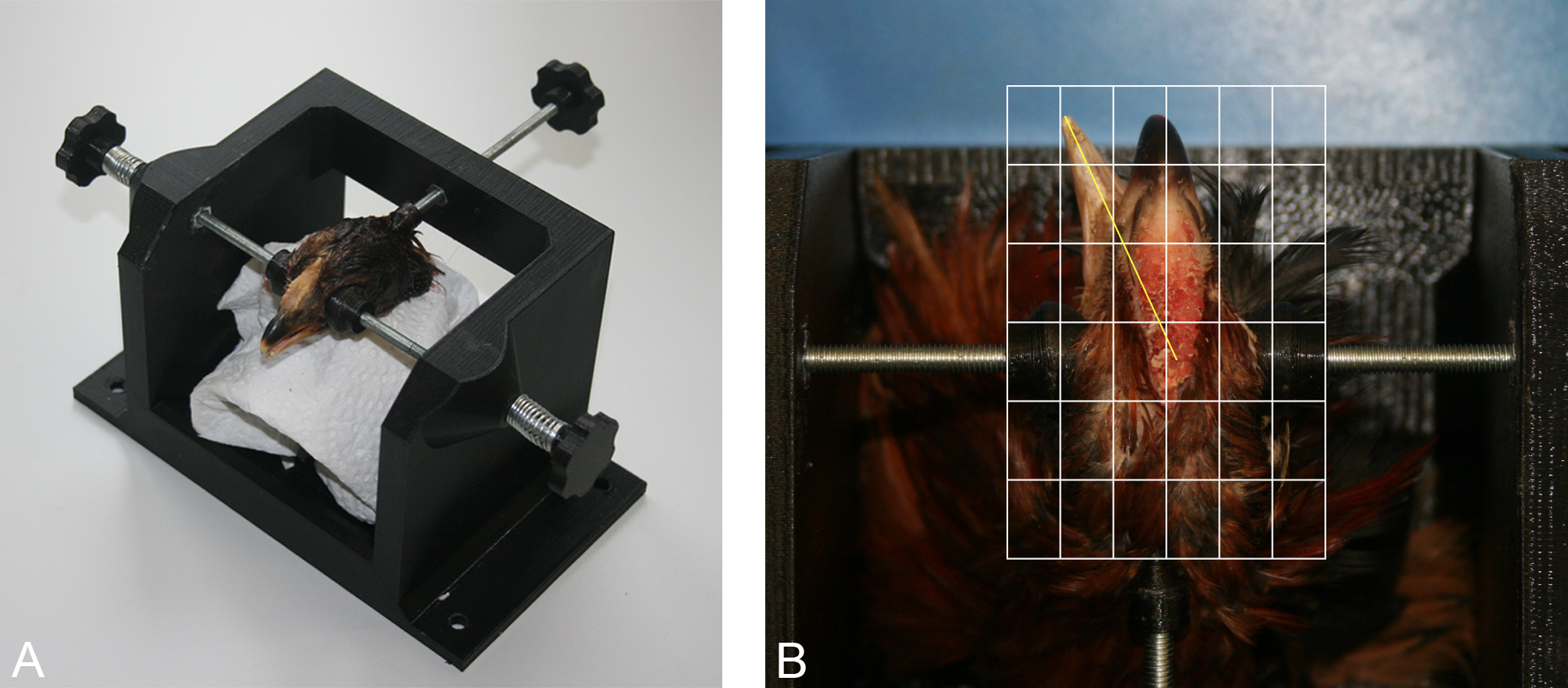

Supplement: Supplementary file 3 — Phenotyping tool for the measurement of beak angles. The head is fixed between two screws, which sit in the orbita of the skull, and one caudal screw, which supports the caudal part of the head. The photograph is taken from directly above. Based on the photograph, the 0° axis is defined as perpendicular to the two screws and through the median plane of the head (central vertical line of the grid). The axis of the beak is defined by drawing a line along the base and middle part of the upper or lower beak (yellow line). (TIFF 10326 kb) [file 12917_2018_1398_MOESM3_ESM.tif]

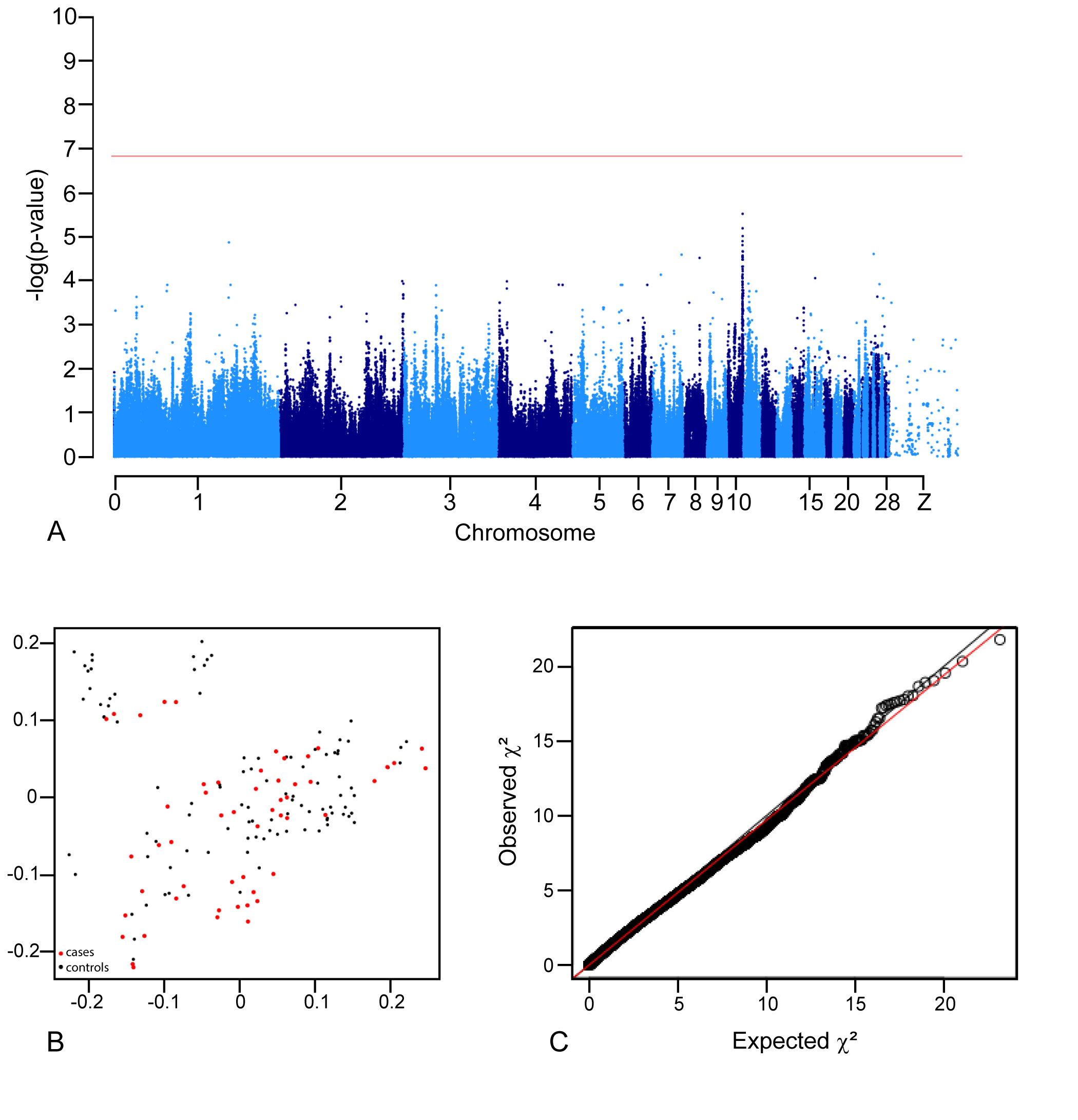

Supplement: Supplementary file 6 — GWAS of 53 cases and 102 controls. (A) Manhattan plot. The red line marks the 5% Bonferroni-corrected threshold for 341,115 independent tests (pBONF 1.47 × 10− 7). (B) MDS plot. (C) QQ plot. (TIFF 1480 kb) [file 12917_2018_1398_MOESM6_ESM.tif]

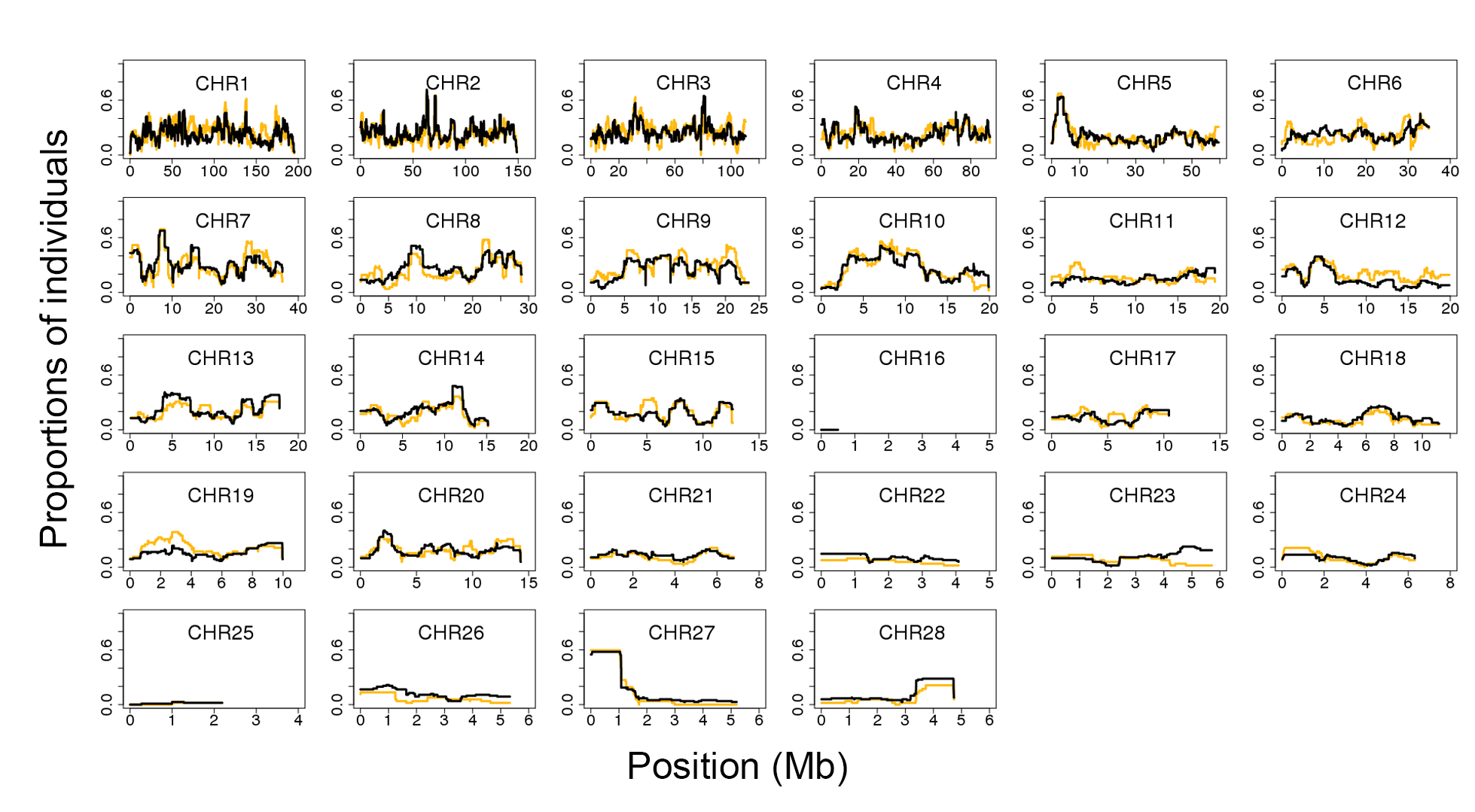

Supplement: Supplementary file 7 — Comparison of runs of homozygosity between cases and controls on the autosomes. Chromosome-wise plots show on the x-axis the mega base (Mb) position on the chromosome (CHR), and on the y-axis the proportions of cases (yellow) and controls (black) being homozygous. (TIFF 1394 kb) [file 12917_2018_1398_MOESM7_ESM.tif]
